# Supplementary material for: Vitamin D Modified DSS-Induced Colitis in Mice via STING Signaling Pathway
Source: Biology (Basel). 2025 Jun 18;14(6):715. doi: 10.3390/biology14060715 (PMC12190092; doi:10.3390/biology14060715)
Supplement: Supplementary file 1 [file biology-14-00715-s001.zip › Supplementary Materials S1: 16s-report of gut microbiota/src/content.html]

谷禾信息 结题报告


### 1 项目概述

|  |  |  |  |
| --- | --- | --- | --- |
| 项目名称 | 吴志浩20个粪便样本 | 单位 | 苏州大学 |
| 样本数 | 20个 | 样本类型 | 小鼠粪便样本 |
| 测序平台 | Illumina NovaSeq6000 | 测序区段 | 16S V4区 |

- **示例报告详细解读视频（上）：** http://www.guhejk.com/wordpress/?p=3654
- **示例报告详细解读视频（下）：** http://www.guhejk.com/wordpress/?p=3660

##### 1.1 项目数据分析结果汇总说明

- **分组样本元信息情况：** 各组样本数量均衡。
- **测序质量：** 各样本数据量相对均一，在10万reads左右。
- **ASV数量情况：** ASV数量平均在400-800左右，相对较多。
- **Alpha多样性：** 各组间alpha多样性存在差异。
- **Beta多样性：** beta多样性组间存在显著差异。
- **物种构成：** 物种构成符合肠道菌群构成特征。
- **组间菌属差异：** 组间菌属差异较多，LEfse结果差异较明显。
- **Bugbase情况：** Bugbase差异部分显著。
- **FAPROTAX：** FAPROTAX结果组间存在显著差异。
- **Picrust2功能预测：** 代谢部分KEGG差异较多。
- **随机森林分组预测：** 基于ASV的分组预测效果较好可以区分各分组。

注：分组及元信息表详情见 metainfo.txt


杭州谷禾信息技术有限公司


---

### 2 技术介绍

微生物多样性测序（扩增子测序）是基于二代高通量测序对16S/18S/ITS等序列进行测序。可以同时检测样本中的
优势物种、稀有物种及一些未知物种的检测，获得样本的微生物群落组成以及相对丰度。

16S rDNA是细菌16S rRNA相对应的DNA序列，存在于所有细菌的基因组中，其中包括保守区域和高变区域，保守
区在细菌间差异不大，高变区在不同的种属间有一定的差异性，可以用做细菌分类鉴定的指标。通过选取特定可
变区域，用保守区段设计引物进行PCR扩增，通过16S rDNA扩增子测序对高变区进行系统鉴定，来研究环境样本
的微生物群落结构组成。

##### 2.1 实验流程

|  |
| --- |
|  |
| Fig 2-1-1 实验流程图 |
|

样本接收后提总DNA，然后对样本进行质检，质检合格后进行PCR扩增。选择某个或某几个变异区域，设计通
用引物扩增。扩增完成后进行电泳检验，PCR产物合格且同批次阳性对照和阴性对照正常。进入混样及文库QC，
最后检测合格的文库进行高通量测序，使用Illumina NovaSeq平台进行测序。得到的测序数据会用于物种鉴定分析。

  |

  

##### 2.2 数据分析流程

Fig 2-2-1 数据分析流程图

  

杭州谷禾信息技术有限公司


---

### 3 测序数据处理及ASVs结果统计

采用 Illumina NovaSeq6000 测序平台进行双端测序得到的下机数据称之为Raw PE。然后进行拼接和质控，根据一定的标准过滤掉低质量数据、接头或PCR错误，得到Clean Tags，即本报告下表中的raw\_tags。**软件平台**：Vsearch V2.22.1

得到的Clean Tags再经过去重复序列，去singleton序列，过滤嵌合体，得到可用于后续分析的有效数据 Effective Tags，即下表中的tagsmatchedASVs。通过UNOISE降噪方法获得ASVs表。**软件平台**：Vsearch V2.22.1

  
数据过滤的标准如下：

- 低质量测序序列的过滤及切除（去除每4bp平均质量Q≤20的读段和低于50bp的reads）；
- 接头序列及无关序列的剔除（Adapter和N）；
- 双端overlap小于完全匹配的5bp；

注：序列长度分布详情见 02\_sequence\_statistic/reads\_length\_distribution.txt

### 4 物种注释及构成

为了得到每个ASV对应的物种分类信息，采用QIIME2训练分类器方法对ASVs代表序列进行分类学分析，得到代表序列的物种注释结果。并在各个分类水平上：**domain**（域），**phylum**（门），**class**（纲），**order**（目），**family**（科），**genus**（属），**species**（种）统计每个样本的群落组成。**软件平台**：qiime2

##### 4.1 所有样本各分类水平组成分析 SILVA138版本

分析报告中，默认选用SILVA138数据库进行比对。

分组统计比较请在各分组结果中查看

以下展示了每个样本门、纲、目、科、属、种所有构成情况。**Taxonomic Level** 可以选择不同的分类水平 **Level 1 ~ Level 7**。该物种丰度柱状图 **软件平台**：qiime2

**文件目录**： 04\_Taxonomic/taxa\_plot/index.html

该柱状图分析用到的数据结果文件。

- 门 构成表文件： 04\_Taxonomic/taxa\_plot/level-2.csv
- 纲 构成表文件： 04\_Taxonomic/taxa\_plot/level-3.csv
- 目 构成表文件： 04\_Taxonomic/taxa\_plot/level-4.csv
- 科 构成表文件： 04\_Taxonomic/taxa\_plot/level-5.csv
- 属 构成表文件： 04\_Taxonomic/taxa\_plot/level-6.csv
- 种 构成表文件： 04\_Taxonomic/taxa\_plot/level-7.csv

  

以下为**标准化**到10万reads的物种构成结果文件，可以使用excel打开。

- 门 构成表文件： 04\_Taxonomic/taxa/feature\_table\_L2.txt
- 纲 构成表文件： 04\_Taxonomic/taxa/feature\_table\_L3.txt
- 目 构成表文件： 04\_Taxonomic/taxa/feature\_table\_L4.txt
- 科 构成表文件： 04\_Taxonomic/taxa/feature\_table\_L5.txt
- 属 构成表文件： 04\_Taxonomic/taxa/feature\_table\_L6.txt
- 种 构成表文件： 04\_Taxonomic/taxa/feature\_table\_L7.txt
- ASVs 表文件： 01\_pick\_otu/otu-table100k-tax.txt

  

以下为**百分比**的物种构成结果文件，以小数表示，可以使用excel打开。

- 门 构成表文件： 04\_Taxonomic/relative/feature\_table\_L2.txt
- 纲 构成表文件： 04\_Taxonomic/relative/feature\_table\_L3.txt
- 目 构成表文件： 04\_Taxonomic/relative/feature\_table\_L4.txt
- 科 构成表文件： 04\_Taxonomic/relative/feature\_table\_L5.txt
- 属 构成表文件： 04\_Taxonomic/relative/feature\_table\_L6.txt
- 种 构成表文件： 04\_Taxonomic/relative/feature\_table\_L7.txt

  
各分类水平的微生物物种构成汇总统计表： 04\_Taxonomic/relative/Otu\_all\_level/OTU\_summary.csv
  
所有ASVs对应的silva v138版本物种注释结果文件： 04\_Taxonomic/taxonexported/taxonomy.tsv

##### 4.2 greengene 13\_8版本物种注释结果文件

  

- ASVs 表文件： 01\_pick\_otu/gg\_13-8table100k-tax.txt

杭州谷禾信息技术有限公司


---

### 5 多样性分布结果

##### 5.1 Alpha多样性结果

分组统计比较请在各分组分析结果中查看

alpha多样性分析，命令如下：qiime diversity alpha，分别计算获得simpson，ace，shannon，chao1以及goods\_coverage。**软件平台**：qiime2

α多样性是对单个样本中的物种多样性分析，通过一系列统计学指数来评估菌群物种的丰富度（richness）和多样性（diversity）。其中丰富度是衡量单个样本中物种的种类个数，通过分类单位的个数来衡量，多样性指数是衡量群落的异质性。

计算**群落丰富度**（Community richness）的指数:

**Chao1**：用Chao1算法估计样本中所含ASV数目的指数，通过计算群落中只检测到1次和2次的ASV数估计群落中实际存在的物种数。Chao1在生态学中常用来估计物种总数，由Chao(1984)最早提出。

**ACE**：用来估计群落中含有ASV数目的指数，预设将序列量10以下的ASV都计算在内，从而估计群落中实际存在的物种数。是生态学中估计物种总数的常用指数之一，与Chao1算法不同。

计算**群落多样性**（Community diversity）的指数：

**Shannon**：香农-威纳指数综合考虑了群落的丰富度和均匀度。Shannon指数值越高，表明群落的多样性越高。

**Simpson**：辛普森多样性指数对菌群多样性评估，Simpson指数值越高，表明群落多样性越高。一般而言，Shannon指数侧重对群落的丰富度以及稀有ASV，而Simpson指数侧重均匀度和群落中的优势ASV。

  

下表统计了每个样本的各项alpha多样性指标。


##### Alpha多样性不同指数的稀释曲线结果

稀释曲线是从每个样本中随机抽取一定数量的序列，统计这些序列所代表的ASV数目，以随机抽取的序列数与ASV数量来构建曲线。可以用来比较不同样本中的物种多样性，也可以用来说明样本出测序数据量是否足以反映环境中的物种多样性。

样品物种丰度 Alpha 多样性指数稀释曲线图，横坐标表示抽取 reads 数量，纵坐标表示相应 Alpha 多样性指数的的值，图中一个颜色代表一个样本，或一组。测序条数不能覆盖样本时，曲线呈上升趋势， 当曲线趋向平坦时，说明测序数据量足够大，可以反映样品中绝大多数的微生物物种信息。**软件平台：**使用R（version 3.6.3）软件作图。

结果在文件夹:/03\_diversity-metrics/alpha\_rarefaction下

- observed\_otus
- shannon
- faith\_pd

|  |  |  |
| --- | --- | --- |
| | |  |

|  |  |  |
| --- | --- | --- |
| | |  |

|  |  |  |
| --- | --- | --- |
| | |  |

Fig 5-1 稀释曲线图

### 6 分组统计分析结果

杭州谷禾信息技术有限公司


---

### 7 个性化分析补充文件

QIIME2数据文件目录

STAMP数据文件目录

MicrobiomeAnalysis.ca数据文件目录

杭州谷禾信息技术有限公司


---

### 8 参考文献

*- Yongxin Liu, Yuan Qin, Xiaoxuan Guo, Yang Bai. Methods and applications for microbiome data analysis. Hereditas(Beijing)2019/9/2
- Shifu Chen, Yanqing Zhou, Yaru Chen, Jia Gu. fastp: an ultra-fast all-in-one FASTQ preprocessor. Bioinformatics, Volume 34, Issue 17, 01 September 2018, Pages i884–i890
- Steven R. Gill et al. Metagenomic Analysis of the Human Distal Gut Microbiome. Science 02 Jun 2006
- P Ewels, M Magnusson, S Lundin, M Käller. MultiQC: summarize analysis results for multiple tools and samples in a single report. Bioinformatics [Internet]. 2016; 32: 3047–8
- A Gurevich, V Saveliev, N Vyahhi, G Tesler. QUAST: quality assessment tool for genome assemblies. Bioinformatics, 2013
- Parks DH, Imelfort M, Skennerton CT, Hugenholtz P, Tyson GW. 2015. CheckM: assessing the quality of microbial genomes recovered from isolates, single cells, and metagenomes. Genome Research, 25: 1043–1055
- Nicola Segata et al. Metagenomic biomarker discovery and explanation. Curtis Huttenhower Genome Biology, 12:R60, 2011
- Lu J, Breitwieser FP, Thielen P, Salzberg SL. (2017) Bracken: estimating species abundance in metagenomics data. PeerJ Computer Science 3:e104, doi:10.7717/peerj-cs.104
- Ondov BD, Bergman NH, and Phillippy AM. Interactive metagenomic visualization in a Web browser. BMC Bioinformatics. 2011 Sep 30; 12(1):385
- Li, D., Liu, C-M., Luo, R., Sadakane, K., and Lam, T-W., (2015) MEGAHIT: An ultra-fast single-node solution for large and complex metagenomics assembly via succinct de Bruijn graph. Bioinformatics, doi: 10.1093/bioinformatics/btv033 [PMID: 25609793]
- Buchfink B., Xie C. and Huson D.H. Fast and sensitive protein alignment using DIAMOND. Nat Methods, 2015;12(1):59-60
- Fu L., et al. CD-HIT: accelerated for clustering the next-generation sequencing data.Bioinformatics, 2012;28(23): 3150-3152
- Langmead B, Salzberg S. Fast gapped-read alignment with Bowtie 2. Nature Methods. 2012, 9:357-359
- Li H, Handsaker B, Wysoker A, Fennell T, Ruan J, Homer N, Marth G, Abecasis G, Durbin R, and 1000 Genome Project Data Processing Subgroup, The Sequence alignment/map (SAM) format and SAMtools, Bioinformatics (2009) 25(16) 2078-9 [19505943]
- Doug Hyatt et al. Prodigal: prokaryotic gene recognition and translation initiation site identification. BMC Bioinformatics volume 11, Article number: 119 (2010)
- Patro, R., Duggal, G., Love, M. I., Irizarry, R. A., & Kingsford, C. (2017). Salmon provides fast and bias-aware quantification of transcript expression. Nature Methods
- Fast genome-wide functional annotation through orthology assignment by eggNOG-mapper. Jaime Huerta-Cepas, Damian Szklarczyk, Lars Juhl Jensen,Christian von Mering and Peer Bork. Submitted (2016)
- Alcock et al. 2019. CARD 2020: antibiotic resistome surveillance with the comprehensive antibiotic resistance database. Nucleic Acids Research, gkz935. [Epub ahead of print] [PMID 31665441]
- Liu B, Zheng DD, Jin Q, Chen LH and Yang J, 2019. VFDB 2019: a comparative pathogenomic platform with an interactive web interface. Nucleic Acids Res. 47(D1):D687-D692
- Chen LH, Zheng DD, Liu B, Yang J and Jin Q, 2016. VFDB 2016: hierarchical and refined dataset for big data analysis-10 years on. Nucleic Acids Res. 44(D1):D694-D697
- Kanehisa M., et al., The KEGG resource for deciphering the genome. Nucleic acids research,2004;32(suppl 1): D277-D280
- Yuki Moriya et al. KAAS: an automatic genome annotation and pathway reconstruction server. Nucleic Acids Res. 2007 Jul; 35(Web Server issue): W182–W185
- Dongwan Kang et al. MetaBAT 2: an adaptive binning algorithm for robust and efficient genome reconstruction from metagenome assemblies. PeerJ Preprints
- Gherman V. Uritskiy et al. MetaWRAP—a flexible pipeline for genome-resolved metagenomic data analysis. Microbiome volume 6, Article number: 158 (2018)
- Martin Urban, Alayne Cuzick, James Seager, Valerie Wood, Kim Rutherford,Shilpa Yagwakote Venkatesh et al. PHI-base: the pathogen–host interactions database. Nucleic Acids Research, Volume 48, Issue D1, 08 January 2020, Pages D613–D620
- Milton H. Saier, Jr, Vamsee S. Reddy, Brian V. Tsu, Muhammad Saad Ahmed,Chun Li, Gabriel Moreno-Hagelsieb. The Transporter Classification Database (TCDB): recent advances. Nucleic Acids Research, Volume 44, Issue D1, 4 January 2016, Pages D372–D379
- Overbeek R, Begley T, Butler RM, Choudhuri JV, Chuang HY, Cohoon M, de Crecy-Lagard V, Diaz N, Disz T, Edwards R, et al. The subsystems approach to genome annotation and its use in the project to annotate 1000 genomes. Nucleic Acids Res. 2005;33:5691–5702
- Caner Bağcı.Sina Beier.Anna Górska.Daniel H. Huson. Introduction to the Analysis of Environmental Sequences: Metagenomics with MEGAN. Evolutionary Genomics pp 591-604*

杭州谷禾信息技术有限公司


---

### 9 目录结构

```
.
├── 01_pick_otu -> ASV聚类分析及过滤
│   └── summary -> ASV统计结果目录
│    │  ├── rawotu-summary.txt -> 测序reads总数、均值及ASV数量分布
│    ├── rep_set.fna -> ASV代表序列（fasta格式）
│    ├── repseq.qza -> ASV代表序列（qiime2的qva格式）
│    ├── rawotu_table.biom -> 原始序列生成的ASV计数表（biom文件）
│    ├── rawotu_table.txt -> 原始序列生成的ASV计数表（文本表文件）
│    ├── otu-table100k-tax.biom -> 标准化到10万reads的ASV计数表-带物种注释（biom文件）
│    ├── otu-table100k-tax.txt -> 标准化到10万reads的ASV计数表-带物种注释（文本表文件）
│    ├── otu-filter-minfeq-tax.biom -> 过滤低于万分之一reads的标准化ASV计数表-带物种注释（biom文件）
│    ├── otu-filter-minfeq-tax.txt -> 过滤低于万分之一reads的标准化ASV计数表-带物种注释（文本表文件）
│    └── gg_13-8table100k-tax.biom -> 标准化到10万reads的greengene13_8数据库计数表-带物种注释（biom文件）
├── 02_sequence_statistic -> 序列长度及个样本分组数据量统计
│   ├── reads_length_distribution.txt -> 测序序列读长
│   ├── Group_Sample_stats_OTU.pdf -> 各分组ASV数量及reads数
│   └── Sample_stats_OTU.pdf -> 各样本ASV数量及reads数
├── 03_diversity-metrics -> alpha及beta多样性分析值
│   └── alpha -> alpha多样性计算目录
│   └── XXX_emperor -> beta多样性qiime2导出目录，可进入目录点击index.html
│   │  ├── alpha_div.txt -> 样本alpha多样性表
│   ├── rooted-tree.nwk -> 构建的ASV进化树
│   ├── XXXX_dm.txt -> beta多样性不同XXX算法的距离矩阵
│   └── XXXX_pc.txt -> beta多样性不同XXX算法的ordination降维特征
├── 04_Taxonomic -> 物种注释结果目录
│   ├── silva138taxonomy.qza -> 基于silva138的菌属物种注释（qiime2的qza文件）
│   ├── relative -> 相对丰度百分比物种构成表，L2-L7分别是门、纲、目、科、属、种
│   ├── taxa -> 标准化到10万reads的物种构成表，L2-L7分别是门、纲、目、科、属、种
│   └── taxa_plot -> qiime2导出物种构成丰度表及图，可以打开目录下的index.html，csv为excel格式表
├── Picrust2 -> 基因功能及代谢通路注释结果目录，来自Picrust2.3
│   ├── KO_metagenome_out -> KEGG基因注释结果，KOpred_metagenome_unstrat_descr.tsv为包含基因名称的丰度表
│   ├── EC_metagenome_out -> 酶注释结果，ECpred_metagenome_unstrat_descr.tsv为包含基因名称的丰度表
│   ├── KEGGpathways_out -> KEGG代谢通路注释结果，KEGGpath_abun_unstrat_descr.tsv为包含代谢通路的丰度表
│   ├── pathways_out -> METACYC代谢通路注释结果，METACYCpath_abun_unstrat_descr.tsv为包含代谢通路的丰度表
│   ├── GMMmodelout -> 菌群代谢产物模块预测结果，modules.tsv为丰度表
│   ├── GBMmodelout -> 菌群神经递质代谢产物模块预测结果，modules.tsv为丰度表
│   ├── CAZYout -> 菌群碳水化合物代谢CAZy预测结果，pred_metagenome_unstrat.tsv为丰度表
│   ├── COG_metagenome_out -> COG预测，COGpred_metagenome_unstrat_descr.tsv为丰度表
│   ├── PFAM_metagenome_out -> PFAM功能域模块预测结果，pred_metagenome_unstrat.tsv为丰度表
│   ├── TIGRFAM_metagenome_out -> TIGRFAM功能域模块预测结果，pred_metagenome_unstrat.tsv为丰度表
│   ├── GBMmodelout -> 菌群神经递质代谢产物模块预测结果，modules.tsv为丰度表
│   ├── GBMmodelout -> 菌群神经递质代谢产物模块预测结果，modules.tsv为丰度表
│   ├── GBMmodelout -> 菌群神经递质代谢产物模块预测结果，modules.tsv为丰度表
│   ├── GBMmodelout -> 菌群神经递质代谢产物模块预测结果，modules.tsv为丰度表
│   └── Unigenes.RPKM.xls -> 每个基因在每个样本中的RPKM
├── STAMP -> 用于STAMP软件进行分析所需文件
├── Groups -> 不同分组情况下的统计结果目录
│   ├── alphadiv  -> alpha多样性统计结果
│   ├── betadiv -> beta多样性统计结果
│   │   ├── pca_analysis -> PCA分析结果
│   │   ├── pcoa_bray_analysis -> PCOA分析结果
│   │   ├── pcoa_unweighted_unifrac_analysis -> PCOA非加权
│   │   ├── pcoa_weighted_unifrac_analysis -> PCOA加权
│   │   └── nmds_analysis -> NMDS分析结果，Envfit统计结果查看Envfit.result.log
│   ├── otuVenn  -> ASV各组韦恩图
│   ├── taxanomyBar  -> 各样本及分组物种构成柱状图
│   │   ├── taxon_hist -> include_all\all为显示top10，其他所有归为other；include_all\top10为仅计算top10，不包含其他；without_unknown为去除了未分类的分类单元
│   │   └── taxon_hist_group -> 为各个分组的均值的物种构成柱状图，目录同上。
│   ├── Network  -> 物种网络图，提供了门层面的关联网络
│   ├── meta  -> 元信息统计检验
│   ├── Lefse_Analysis  -> Lefse统计结果，biomarkers_raw_images目录包含具体菌属的标志图
│   ├── Bugbase -> Bugbase分析结果，BugBase_pvlaue.txt为统计检验pvalue
│   │   ├── normalized_otus -> 根据拷贝数标准化ASV
│   │   ├── otu_contributions -> 各ASV对Bugbase各特征的贡献
│   │   ├── predicted_phenotypes -> Bugbase各特征的样本评估结果
│   │   ├── pcoa_weighted_unifrac_analysis -> PCOA加权
│   │   └── nmds_analysis -> NMDS分析结果，Envfit统计结果查看Envfit.result.log
│   ├── FAPROTAX -> FAPROTAX预测及分析结果
│   │   ├── FAPROTAX_heatmap -> FAPROTAX丰度状况，具体ASV构成查看:FAPROTAX_report.txt
│   │   ├── Markers -> 组间差异统计检验
│   │   ├── pcoa_unweighted_unifrac_analysis -> PCOA非加权
│   │   ├── pcoa_weighted_unifrac_analysis -> PCOA加权
│   │   └── nmds_analysis -> NMDS分析结果，Envfit统计结果查看Envfit.result.log
│   └── diff_analysis -> 组间统计检验及差异分析结果
│       ├── Anosim_analysis -> Anosim分析结果
│       ├── metagenomeRXXXX -> 使用metagenomeSeq进行统计检验结果
│       ├── UnivarTestXXXX -> 使用非参数检验进行统计检验结果
│       ├── TukeyHSD -> 使用Tukey进行统计检验结果
│       ├── TaxaMarkers -> 物种差异分析结果
│       ├── Correlation -> 基于metagenomeSeq的差异指标相互相关性分析结果
│       ├── heatmap -> 显著差异指标的热图
│       ├── metaCorrelation -> 差异指标与元数据及环境因子的相关性分析结果
│       ├── diff_filter -> 统计差异指标筛选后的数据表
│       └── RF -> 使用随机森林进行模型构建和预测结果
└─ 
```

杭州谷禾信息技术有限公司


---

### 10 附录

- 问题解答
- 软件下载
- 数据库信息

##### 10.1 英文Methods

英文方法

##### 10.2 文章引用与致谢

如果您的研究课题使用了杭州谷禾信息技术有限公司的测序和分析服务，我们期望您在论文发表时，在Method部分或Acknowledgements部分引用或提及杭州谷禾信息技术有限公司。
  

杭州谷禾信息技术有限公司


---

### 帮助文档
